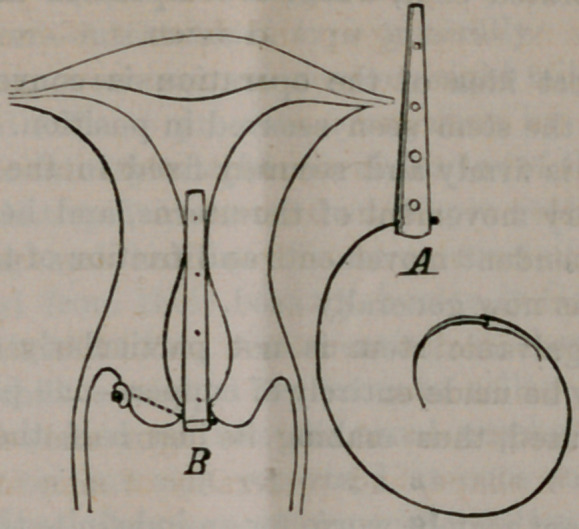# New Intra-Uterine Pessary

**Published:** 1877-04

**Authors:** V. H. Taliaferro

**Affiliations:** Atlanta, Ga.; Professor of Obstetrics and Diseases of Women in the Atlanta Medical College; President of the Atlanta Academy of Medicine; Secretary of the Board of Health of the State of Georgia


ATLANTA

MEDICAL AND SURGICAL JOURNAL

VOL. XV.] APRIL—1877. [No. 1

### Original Communications.

### NEW INTRA-UTERINE PESSARY, WITH NOVEL METHOD OF SECURING IT IN POSITION.

BY V. H. TALIAFERRO, M.D., ATLANTA, GA.
Professor of Obstetrics and Diseases of Women in the Atlanta Medical College; President of the Atlanta Academy of Medicine; Secretary of the Board of Health of the State of Georgia.

The objectionable features of the intra-uterine pessaries, are their tendency to excite inflammatory action in the uterine and peri-uterine structures, and the difficulty in retaining them in position. With these overcome, the intra-uterine stem is an instrument of great value, and applicable to a variety of conditions not amenable to other resources, as so often found in flexions, superinvolution, amenorrhœa, sterility, etc.

The rather bulky instruments of Simpson, Kiwisch, and Valliex, adopted with great enthusiasm by their illustrious authors, yielded such unfavorable results as to bring them into much disfavor, and to well nigh cause, for the time being, their entire abandonment.

Of late years, instruments of a more approved construction, together, doubtless, with our greater aptitude at diagnosis, and our better understanding of the contraindications to their use, have contributed, no doubt, to their present popularity with many gynæcologists.

The special points to be observed in the use of the intra-uterine stem are:

1st. A fit sufficiently loose to make no undue pressure at any point, and which at the same time is sufficiently close and secure in position to prevent any free motion of the instrument.

2d. It should be perfectly secured in its position in the cervix, so that no slipping up and down is allowable.

3d. It should cause no pain or uneasiness, either in its introduction or its continued use.

4th. The point of the instrument, when introduced, should extend but little beyond the internal os.

5th. The uterus should be free from any marked tenderness or congestion.

6th. The peri-uterine structures should be soft and elastic, and free from tenderness.

7th. The instrument should be light and simple in construction, flat in shape, and preferably of zinc and copper, or copper alone.

One of the chief difficulties in the use of the intra-uterine stem is to retain it in the uterus. When the uterine canal has its normal axis, any of the ordinary stems with vaginal bulb are readily retained, as the instrument abuts upon the posterior vaginal wall. If, however, as so frequently occurs in displacements, the uterine axis is brought more nearly in coincidence with that of the vagina, then the support of the posterior vaginal wall is gone, and the instrument slips from the uterus into the vagina. Various devices have been resorted to for the purpose of meeting this trouble, all of which I think more or less objectionable. Those with expanding arms, whether of metal or rubber, are most reprehensible.

Sir J. Y. Simpson used the lever pessary for the purpose of directing the os back upon the posterior vaginal wall, upon which the instrument would rest. Prof. Thomas, of New York, uses the lever with a rubber diaphragm as a support for the stem. These, while far preferable to all the means heretofore proposed for retaining the stem in position, are yet objectionable in many cases where it is not desirable to subject the uterus to the double stimulation of a vaginal and intra-uterine pessary. They are again open to the objection that the stem is not *fixed* in its position, and hence capable of a constant upward and downward play, from the almost incessant physiological movemnts of the uterus.

In the simple stem here offered, with the method of securing it, I have endeavored to meet the indications and difficulties mentioned.

The stem, as represented in the wood-cut, with silver wire and needle attached, is made of strips of sheet zinc and copper, such as obtained from the tin-shops, trimmed with stout scissors to any size desired, and the separate strips rivited together. They can be made in any doctor's shop in a little while. The stem is flattened antero-posteriorly, and adapts itself perfectly to the uterine canal without change of the normal relation (antero-posterior approximation) of the walls of the uterus.

The instrument is slightly galvanic, and hence therapeutic as well as mechanical in its action. As will be observed by the wood-cut, its proximal end has secured to it a delicate silver wire, with small curved needle attached. The wire is fixed to the stem by passing it between the zinc and copper plates, and secured with a compressed shot.

To apply the pessary, the patient is placed before a good light, in the semi-prone position, and the uterus exposed with Sims' speculum. The anterior lip of the os is then seized with a tenaculum, and the uterus straightened out in the vaginal axis and steadied, while, with suitable needle forceps, the needle—already attached by delicate silver wire to the stem—is made to pass immediately within the os and out laterally through the cervix, emerging just below the vaginal junction. The point of the needle is then grasped with the forceps and withdrawn. The stem is introduced in the cervix while the wire is being at the same time withdrawn, until both are in position. A small lead button is slipped down the wire, and on this a perforated shot, which is compressed and the wire cut close.

A very correct idea of the operation is conveyed by the wood-cut, with the stem seen secured in position. The stem, as will be seen, is firmly and securely fixed in the cervix, and partakes of every movement of the uterns, and hence incapable of the independent movements and friction of the ordinary *bulb stem* such as now generally used.

When the galvanic stem is not particularly desired, the instrument may be made entirely of copper—one plate instead of two being used, thus making it just half the bulk and weight.

The instrument may be worn, for an indefinite time, without risk of damage or inconvenience to the patient. While using them I direct that, after a few days from the introduction, no check be put upon exercise, either on foot or horseback. The same precautions as to dress are applicable here as in the use of vaginal pessaries, that the uterus may not be forced upon the floor of the pelvis by the pressure of tight clothes or corsets, and subjected to friction and concussions. The vaginal douche, of hot water, should be daily used while the pessary is worn.

## Figures and Tables

**Figure f1:**